# Method of primary breast cancer detection and the disease-free interval, adjusting for lead time

**DOI:** 10.1093/jnci/djad230

**Published:** 2023-11-03

**Authors:** Linda de Munck, Anouk H Eijkelboom, Johannes D M Otten, Mireille J M Broeders, Sabine Siesling

**Affiliations:** Department of Research and Development, Netherlands Comprehensive Cancer Organisation, Utrecht, the Netherlands; Department of Research and Development, Netherlands Comprehensive Cancer Organisation, Utrecht, the Netherlands; Department of Health Technology and Services Research, Technical Medical Centre, University of Twente, Enschede, the Netherlands; Department for Health Evidence, Radboud University Medical Center, Nijmegen, the Netherlands; Department for Health Evidence, Radboud University Medical Center, Nijmegen, the Netherlands; Dutch Expert Centre for Screening, Nijmegen, the Netherlands; Department of Research and Development, Netherlands Comprehensive Cancer Organisation, Utrecht, the Netherlands; Department of Health Technology and Services Research, Technical Medical Centre, University of Twente, Enschede, the Netherlands

## Abstract

**Background:**

Little is known about the impact of screen-detected breast cancer compared with clinically detected breast cancer on the disease-free interval (ie, free from locoregional recurrences, distant metastasis, contralateral breast cancer). Moreover, it is thought that most studies overestimate the beneficial effect of screening, as they do not adjust for lead time. We investigated the association between method of breast cancer detection and disease-free interval, taking lead time into account.

**Methods:**

Women aged 50-76 years, diagnosed with breast cancer between 2005 and 2008 were selected from the Netherlands Cancer Registry. Women diagnosed in 2005 were divided into screen-detected and clinically detected cancer and had a follow-up of 10 years (2005 cohort). Women diagnosed in 2006-2008 were divided into screen-detected, interval, and nonscreen-related cancer and had a follow-up of 5 years (2006-2008 cohort). A previously published method was used to adjust for lead time. Analyses were repeated correcting for confounding variables instead of lead time.

**Results:**

The 2005 cohort included 6215 women. Women with screen-detected cancer had an improved disease-free interval compared with women with clinically detected cancer (hazard ratio [HR] = 0.77, 95% confidence interval [CI] = 0.68 to 0.87). The 2006-2008 cohort included 15 176 women. Women with screen-detected or interval cancer had an improved disease-free interval compared with women with nonscreen-related cancer (HR = 0.76, 95% CI = 0.66 to 0.88; HR = 0.88, 95% CI = 0.78 to 0.99, respectively). Correcting for confounders instead of lead time did not change associations.

**Conclusion:**

Women with screen-detected cancer had an improved disease-free interval compared with women with a nonscreen-related or clinically detected cancer, after correction for lead time.

Previous studies have shown that women with screen-detected breast cancer have a longer overall as well as disease-free survival compared with women with clinically detected breast cancer (1-5). However, these studies are susceptible for lead time and length time bias, possibly leading to an overestimation of the beneficial effect of screening. It has been argued that this artificial overestimation is the main reason for favorable survival, and therefore detection method of the tumor is currently not used to estimate the risk of cancer recurrence or overall survival (6,7).

Screening studies are susceptible for 2 types of biases. First, length time is introduced because more slowly growing tumors have a longer presymptomatic screen-detectable phase and are therefore more likely to be screen-detected. Overdiagnosis, the most extreme form of length time bias, means that the patient is diagnosed with a tumor that would not have been diagnosed in the absence of screening. Second, lead time can be defined as the time between the date of detection of a screen-detected cancer and the date it would have been diagnosed without screening. A method to correct for lead time has been described by Duffy et al. (8). Studies using this method, hereafter referred to as the Duffy method, found an improved breast cancer-specific survival for patients with a screen-detected cancer compared with patients with an interval breast cancer (a tumor diagnosed after a negative screening result) (9-11). Another study, adjusting for confounders such as tumor stage, subtype, and grade, as well as adjusting for lead time, found no difference in survival between patients with a screen-detected or clinically detected cancer (12).

Tumor characteristics are usually used as confounding variables in analyses concerning the method of detection and overall and disease-free survival. One might argue that correcting for patient and tumor characteristics can be seen as a (although not perfect) proxy for correcting for lead time. It would be interesting to perform analyses correcting for lead time and correcting for confounders separately, using the same study population, and to compare the results.

The few studies taking lead time into account only investigated the association between the method of detection and (breast cancer–specific) survival. However, as the survival of patients with breast cancer is improving, more patients are at risk of developing recurrent disease. Therefore, more knowledge on recurrent disease is desired. The disease-free interval (ie, the period of time between the primary tumor and recurrent disease) would be a suitable endpoint in studies into recurrent diseases.

This study aimed to investigate the association between method of breast cancer detection and the disease-free interval, taking lead time into account. Analyses were repeated correcting for confounding variables (patient and tumor characteristics) only. We hypothesized that screen-detected cancers would have an improved disease-free interval compared with clinically detected cancers after correction for lead time, as well as after correction for confounding.

## Methods

### Study population and data collection

Patients were selected from the Netherlands Cancer Registry (NCR). The NCR is a nationwide population-based cancer registry, hosted by the Netherlands Comprehensive Cancer Organisation, that includes almost all newly diagnosed cancer patients since 1989. Notifications of newly diagnosed tumors are obtained from the nationwide network and registry of histo- and cytopathology in the Netherlands (PALGA). Trained data managers subsequently register information on patient, tumor, and treatment characteristics. For breast cancer patients diagnosed in 2005, additional data on locoregional recurrences or distant metastases diagnosed within 10 years following primary tumor diagnosis were available, as these data were obtained from patient files during previous projects. For patients diagnosed in 2006-2008, data on locoregional recurrences or distant metastases occurring within 5 years following diagnosis were available from previous projects. Data on vital status were obtained by linkage to the Municipal Personal Records database. The study was approved by the privacy review board of the NCR. According to the Dutch Central Committee on Research involving Human Subjects, no ethical approval is needed for this study, as it is a retrospective study, which uses data from the NCR.

**Figure 1.**
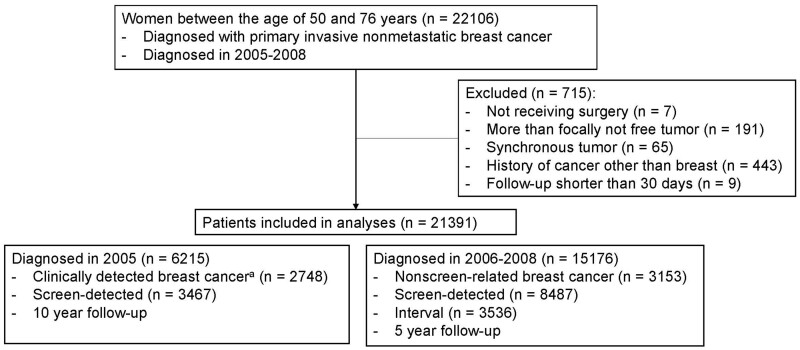
Flowchart of included participants. ^a^Clinically detected breast cancer includes nonscreen-related and interval breast cancer.

Women aged between 50 and 76 years; diagnosed with invasive nonmetastatic breast cancer between January 1, 2005, and December 31, 2008; and surgically treated in a Dutch hospital were selected (see Figure 1). Patients were ineligible if they had been diagnosed with a previous malignant tumor (breast or other localization) in the past 5 years or with a synchronous breast tumor (diagnosed within 30 days of each other); developed a locoregional recurrence, distant metastasis, or contralateral breast tumor within 30 days after diagnosis; died within 30 days after diagnosis; or had a macroscopic residue after surgery or a microscopic residue without adjuvant treatment.

Patients were divided into 2 cohorts based on data availability. The 2005 cohort contains data of patients diagnosed between January 1, 2005, and December 31, 2005. The 2006-2008 cohort contains data of patients diagnosed between January 1, 2006, and December 31, 2008. Data of patients in this latter cohort had been linked previously on an individual level to the data of the Netherlands Breast Cancer Screening Programme so data on method of detection was available. This population-based program has been operational since 1990, initially inviting women aged 49-69 years for a biennial screening examination. From 1998 onward, the age range expanded to 74 years (13). Permission for use of the data was requested from women when they attended screening. This was based on an opt-out option, which was used by 0.02% of all women screened (14).

### Definitions

Women diagnosed in 2005 were divided into 2 detection groups: screen-detected vs clinically detected cancer. A screen-detected cancer was defined as a tumor diagnosed within 24 months after a positive screening result. A clinically detected cancer was defined as a tumor not detected by the screening program. For women diagnosed between 2006 and 2008, a more detailed definition of method of detection was derived: screen-detected, interval, and nonscreen-related cancer. An interval cancer was defined as a tumor diagnosed within 24 months after a negative screening result. A nonscreen-related cancer was defined as a tumor detected more than 24 months after the last screening (ie, not recently screened) or in a woman who had never attended screening.

The primary outcome of the current study was disease-free interval, defined as the period of time between diagnosis of the primary tumor and diagnosis of the recurrent disease (any locoregional recurrence, distant metastasis, or contralateral invasive breast cancer) or the end of follow-up. A locoregional recurrence was defined as the reappearance of cancer in the ipsilateral breast, chest wall, or axillary or supraclavicular lymph nodes. A distant metastasis was defined as the reappearance of breast cancer at a location other than the breast or regional axillary nodes (15). Patients were censored if they died or at the last date of observation.

A patient’s socioeconomic status (SES) was based on scores assigned to the 4 numbers of each patient’s postal code at the time of diagnosis. These scores, based on mean household income, percentage of inhabitants with a low income, percentage of low educatedness, and percentage of unemployment, were provided by the Netherlands Institute for Social Research at an aggregated level (16). Subsequently, these scores were categorized as high, intermediate, and low SES. Age was categorized into younger than 60 years, 60-69 years, 70 years and older. Tumors were divided into 4 subtypes based on the estrogen receptor, progesterone receptor, and HER2 status: 1) estrogen receptor positive and/or progesterone receptor positive, and HER2 negative; 2) estrogen receptor positive and/or progesterone receptor positive, and HER2 positive; 3) estrogen receptor negative and progesterone receptor negative and HER2 negative; and 4) estrogen receptor negative and progesterone receptor negative and HER2 positive. Tumor size and TNM stage were based on pathological assessment or on clinical assessment if the patient received neoadjuvant therapy (17).

### Statistical analysis

Descriptive statistics were used to summarize baseline characteristics of the total population and the separate subgroups. χ^2^ and Kruskal–Wallis tests were used to compare patient, tumor, and treatment characteristics.

The crude risk of developing recurrent disease was estimated using the Kaplan–Meier method, and detection groups were compared using the log-rank test. Cox proportional hazard regression models were used to estimate hazard ratios (HRs) and 95% confidence intervals (CIs) for the association between method of detection and disease-free interval. Women with a clinically detected cancer (ie, interval and nonscreen-related cancers combined, 2005 cohort) or nonscreen-related cancer (2006-2008 cohort) were used as the reference categories. The Duffy method (8) was used to correct for lead time, using a mean sojourn time of 4.3 years to represent the preclinical screen-detectable period (Supplementary Methods, available online) (18). For the 2005 cohort, this resulted in a corrected follow-up time of 6.1 years (calculated using the following equation: 10-([1-e^-(1/4.3*10)^]/[1/4.3])) for all women with a screen-detected cancer and no recurrent disease after 10 years of follow-up. In the 2006-2008 cohort, the corrected follow-up time of women with a screen-detected cancer and no recurrent disease after 5 years of follow-up was 2.0 years (calculated using the following equation: 5-([1-e^-(1/4.3*5)^]/[1/4.3])). The corrected disease-free interval was used in the Cox models to adjust for lead time. All analyses were adjusted for age at diagnosis (continuous).

Additional analyses were performed correcting for confounders (patient and tumor characteristics) only. The following confounders were added to the Cox regression model: age, SES, histology, tumor grade, multifocality, tumor stage, and subtype. Treatment was not added as a confounder to prevent overcorrection, as the type of treatment is mainly based on patient and tumor characteristics. As these analyses were not corrected for lead time, the maximum follow-up was used. To account for missing values, multiple imputation by chained equations was used to impute these values (19). In 2005, a relatively large percentage of patients had missing values for tumor subtype, because HER2 status had only been routinely collected in the NCR since 2006. Other missing values were related to missing information in the patients’ files and were considered as missing at random. Covariates included in the baseline table were used for imputation. Data were imputed 25 times. Rubin’s rule was subsequently used to pool the estimates and standard errors (20). The validity of the imputed data was checked by comparing the values of the complete cases with the imputed values. The imputed data were used for the analyses. Complete cases analyses were performed for comparison. Scaled Schoenfeld residuals plots were used to test the proportionality assumptions (21).

Sensitivity analyses were performed to investigate whether results were similar for women diagnosed with a screen-detected cancer at or an interval cancer after an initial or subsequent screening examination.

A 2-sided *P* value less than .05 was considered statistically significant. All data were analyzed with STATA version 17 software.

## Results

A total of 6215 women were diagnosed in 2005 of whom 3467 (55.8%) had a screen-detected and 2748 (44.2%) a clinically detected cancer. All baseline characteristics, except multifocality, differed statistically between the detection groups (Table 1). A total of 15 176 women were diagnosed in 2006-2008 of whom 8487 (55.9%) had a screen-detected, 3536 (23.3%) an interval, and 3153 (20.8%) a nonscreen-related cancer. All baseline characteristics differed statistically significantly between the detection groups (Table 2).

**Table 1.** Patient, tumor, and treatment characteristics of the total 2005 cohort and specified by method of detection^a^

Characteristics	Total population No. (%)	Clinically detected cancer No. (%)	Screen-detected cancer No. (%)	*P* ^b^
Patients	6215	2748	3467	
Recurrence				<.001
No	4996 (80.4)	2065 (75.1)	2931 (84.5)	
Yes, locoregional recurrence and/or distant metastasis and/or contralateral breast cancer	1219 (19.6)	683 (24.9)	536 (15.5)	
Age at diagnosis, median (IQR)	61 (55-68)	60 (54-67)	62 (56-68)	<.001
SES				.02
High	1948 (31.3)	906 (33.0)	1042 (30.1)	
Medium	2523 (40.6)	1069 (38.9)	1454 (41.9)	
Low	1744 (28.1)	773 (28.1)	971 (28.0)	
Screening round				—^c^
Initial screen	—^c^	—^c^	342 (9.9)	
Subsequent screen	—^c^	—^c^	3125 (90.1)	
Histology				<.001
Ductal	4947 (79.6)	2127 (77.4)	2820 (81.3)	
Lobular	718 (11.6)	376 (13.7)	342 (9.9)	
Mixed	258 (4.2)	104 (3.8)	154 (4.4)	
Other	292 (4.7)	141 (5.1)	151 (4.4)	
Tumor grade				<.001
1	1454 (25.0)	471 (18.7)	983 (29.9)	
2	2668 (46.0)	1089 (43.2)	1579 (48.0)	
3	1684 (29.0)	958 (38.0)	726 (22.1)	
Unknown	409	230	179	
Multifocality				.09
Yes	5156 (85.2)	2246 (84.3)	2910 (85.9)	
No	896 (14.8)	418 (15.7)	478 (14.1)	
Unknown	163	84	79	
Tumor size, cm				<.001
<2	4108 (67.7)	1358 (51.8)	2750 (79.9)	
2-5	1782 (29.4)	1120 (42.7)	662 (19.2)	
>5	176 (2.9)	146 (5.6)	30 (0.9)	
Unknown	149	124	25	
Positive nodes				<.001
0	4065 (65.8)	1520 (55.8)	2545 (73.6)	
1-3	1515 (24.5)	790 (29.0)	725 (21.0)	
>3	601 (9.7)	415 (15.2)	186 (5.4)	
Unknown	34	23	11	
Tumor stage				<.001
I	3117 (50.2)	934 (34.1)	2183 (63.1)	
II	2347 (37.8)	1274 (46.5)	1073 (31.0)	
III	740 (11.9)	534 (19.5)	206 (6.0)	
Unknown	11	6	5	
Tumor subtype				<.001
ER+ and/or PR+ and HER2-	3756 (74.8)	1539 (67.7)	2217 (80.6)	
ER+ and/or PR+ and HER2+	444 (8.8)	226 (9.9)	218 (7.9)	
ER- and PR- and HER2-	300 (6.0)	184 (8.1)	116 (4.2)	
ER- and PR- and HER2+	524 (10.4)	324 (14.3)	200 (7.3)	
Unknown	1191	475	716	
Type of surgery				<.001
Breast-conserving surgery	3811 (61.3)	1378 (50.1)	2433 (70.2)	
Mastectomy	2404 (38.7)	1370 (49.9)	1034 (29.8)	
Chemotherapy				<.001
No	4369 (70.3)	1587 (57.8)	2782 (80.2)	
Yes	1846 (29.7)	1161 (42.2)	685 (19.8)	
Hormonal therapy				<.001
No	3663 (58.9)	1371 (49.9)	2292 (66.1)	
Yes	2552 (41.1)	1377 (50.1)	1175 (33.9)	
Targeted therapy				<.001
No	5905 (95.0)	2544 (92.6)	3361 (96.9)	
Yes	310 (5.0)	204 (7.4)	106 (3.1)	
Radiotherapy				<.001
No	1834 (29.5)	929 (33.8)	905 (26.1)	
Yes	4381 (70.5)	1819 (66.2)	2562 (73.9)	
Neoadjuvant systemic therapy				<.001
No	6016 (96.8)	2582 (94.0)	3434 (99.0)	
Yes	199 (3.2)	166 (6.0)	33 (1.0)	
Axillary lymph node dissection				<.001
No	3457 (55.6)	1261 (45.9)	2196 (63.3)	
Yes	2758 (44.4)	1487 (54.1)	1271 (36.7)	

a Percentages are calculated on known values only. ER = estrogen receptor; IQR = interquartile range; PR = progesterone receptor; SES = socioeconomic status.

b χ^2^ and Kruskal–Wallis test were used to compare patients in the different method of detection groups. The *P* value is calculated on known values only.

c Not applicable for this subgroup of patients.

**Table 2.** Patient, tumor, and treatment characteristics of the total 2006-2008 cohort and specified by method of detection^a^

Characteristics	Total population No. (%)	Nonscreen-related cancer No. (%)	Screen-detected cancer No. (%)	Interval cancer No. (%)	*P* ^b^
Patients	15 176	3153	8487	3536	
Recurrence					<.001
No	13 468 (88.7)	2642 (83.8)	7802 (91.9)	3024 (85.5)	
Yes, locoregional recurrence and/or distant metastasis and/or contralateral breast cancer)	1708 (11.3)	511 (16.2)	685 (8.1)	512 (14.5)	
Age at diagnosis, median (IQR)	61 (55-68)	61 (54-68)	62 (56-68)	60 (55-66)	<.001
SES					.003
High	4805 (31.7)	970 (30.8)	2660 (31.3)	1175 (33.2)	
Medium	6201 (40.9)	1244 (39.5)	3507 (41.3)	1450 (41.0)	
Low	4170 (27.5)	939 (29.8)	2320 (27.3)	911 (25.8)	
Screening round					<.001
Initial screen	—^c^	—^c^	822 (9.7)	476 (13.5)	
Subsequent screen	—^c^	—^c^	7667 (90.3)	3060 (86.5)	
Histology					<.001
Ductal	12 012 (79.2)	2465 (78.2)	6848 (80.7)	2699 (76.3)	
Lobular	1684 (11.1)	343 (10.9)	856 (10.1)	485 (13.7)	
Mixed	701 (4.6)	147 (4.7)	373 (4.4)	181 (5.1)	
Other	779 (5.1)	198 (6.3)	410 (4.8)	171 (4.8)	
Tumor grade					<.001
1	3750 (26.4)	599 (20.9)	2575 (31.9)	576 (17.7)	
2	6514 (45.9)	1276 (44.5)	3838 (47.5)	1400 (43.1)	
3	3929 (27.7)	991 (34.6)	1663 (20.6)	1275 (39.2)	
Unknown	983	287	411	285	
Multifocality					<.001
Yes	12 729 (85.7)	2588 (84.1)	7216 (86.8)	2925 (84.6)	
No	2119 (14.3)	489 (15.9)	1098 (13.2)	532 (15.4)	
Unknown	328	76	173	79	
Tumor size, cm					<.001
<2	10 101 (68.1)	1560 (52.3)	6737 (80.0)	1804 (52.8)	
2-5	4306 (29.1)	1257 (42.2)	1599 (19.0)	1450 (42.4)	
>5	415 (2.8)	164 (5.5)	87 (1.0)	164 (4.8)	
Unknown	354	172	64	118	
Positive nodes					<.001
0	9821 (65.4)	1702 (54.7)	6225 (74.1)	1894 (53.8)	
1-3	3754 (25.0)	956 (30.7)	1723 (20.5)	1075 (30.5)	
>3	1450 (9.7)	451 (14.5)	449 (5.3)	550 (15.6)	
Unknown	151	44	90	17	
Tumor stage					<.001
I	7716 (50.9)	1100 (34.9)	5407 (63.8)	1209 (34.2)	
II	5664 (37.4)	1444 (45.8)	2572 (30.3)	1648 (46.7)	
III	1776 (11.7)	606 (19.2)	497 (5.9)	673 (19.1)	
Unknown	20	3	11	6	
Tumor subtype					<.001
ER+ and/or PR+ and HER2-	11 020 (77.3)	2146 (72.3)	6 559 (82.8)	2315 (68.9)	
ER+ and/or PR+ and HER2+	1085 (7.6)	253 (8.5)	536 (6.8)	296 (8.8)	
ER- and PR- and HER2-	744 (5.2)	202 (6.8)	294 (3.7)	248 (7.4)	
ER- and PR- and HER2+	1403 (9.8)	367 (12.4)	537 (6.8)	499 (14.9)	
Unknown	924	185	561	178	
Type of surgery					<.001
Breast-conserving surgery	9384 (61.8)	1512 (48.0)	5939 (70.0)	1933 (54.7)	
Mastectomy	5792 (38.2)	1641 (52.0)	2548 (30.0)	1603 (45.3)	
Chemotherapy					<.001
No	10 306 (67.9)	1886 (59.8)	6603 (77.8)	1817 (51.4)	
Yes	4870 (32.1)	1267 (40.2)	1884 (22.2)	1719 (48.6)	
Hormonal therapy					<.001
No	8764 (57.7)	1534 (48.7)	5494 (64.7)	1736 (49.1)	
Yes	6412 (42.3)	1619 (51.3)	2993 (35.3)	1800 (50.9)	
Targeted therapy					<.001
No	14 121 (93.0)	2880 (91.3)	8087 (95.3)	3154 (89.2)	
Yes	1055 (7.0)	273 (8.7)	400 (4.7)	382 (10.8)	
Radiotherapy					<.001
No	4344 (28.6)	1114 (35.3)	2182 (25.7)	1048 (29.6)	
Yes	10 832 (71.4)	2039 (64.7)	6305 (74.3)	2488 (70.4)	
Neoadjuvant systemic therapy					<.001
No	14 524 (95.7)	2865 (90.9)	8361 (98.5)	3298 (93.3)	
Yes	652 (4.3)	288 (9.1)	126 (1.5)	238 (6.7)	
Axillary lymph node dissection					<.001
No	8994 (59.3)	1523 (48.3)	5753 (67.8)	1718 (48.6)	
Yes	6182 (40.7)	1630 (51.7)	2734 (32.2)	1818 (51.4)	

a Percentages are calculated on known values only. ER = estrogen receptor; IQR = interquartile range; PR = progesterone receptor; SES = socioeconomic status.

b χ^2^ and Kruskal–Wallis test were used to compare patients in the different method of detection groups. The *P* value is calculated on known values only.

c Not applicable for this subgroup of patients.

### 2005 cohort

**Figure 2.**
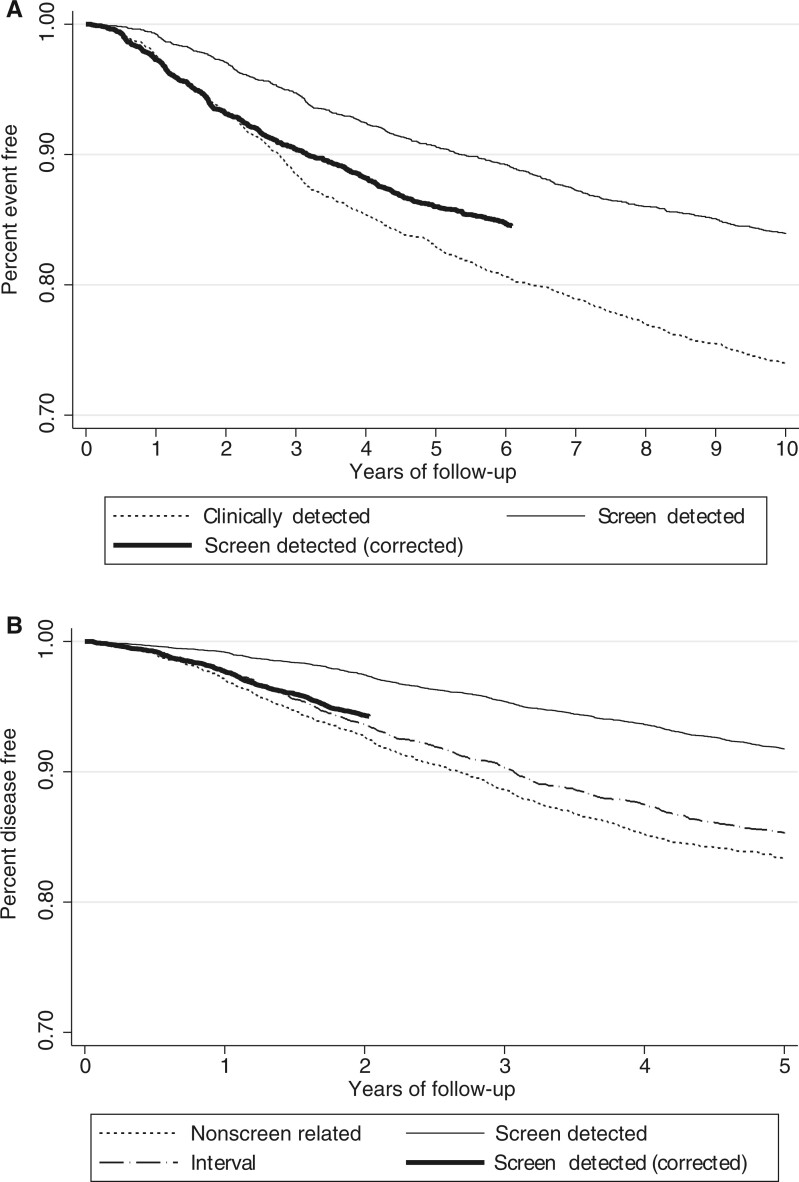
Disease-free interval of women in the 2005 **(A)** and 2006-2008 cohort **(B)**, specified by method of detection and with and without lead time correction. Range on *y*-axis is 0.7-1.0.

During the 10-year follow-up period, 84.5% of the women with a screen-detected cancer had a 10-year disease-free interval compared with 75.1% of the women with a clinically detected cancer (Figure 2, A). After correcting for lead time, 84.7% of the women with a screen-detected cancer had a 6.1-year disease-free interval. To compare, 80.6% of women with a clinically detected cancer had a 6.1-year disease-free interval. The unadjusted analysis showed that women with a screen-detected cancer had an improved 10-year disease-free interval compared with women with a clinically detected cancer (HR = 0.56, 95% CI = 0.50 to 0.63) (Table 3). This effect remained present after correcting for lead time (HR = 0.77, 95% CI = 0.68 to 0.87) or confounders (HR = 0.72, 95% CI = 0.64 to 0.81) (Table 3;Supplementary Table 1, available online). After adjusting for lead time or confounders, women with a screen-detected cancer detected at an initial or subsequent screen had an improved disease-free interval compared with women with a clinically detected tumor (Supplementary Table 2, available online).

**Table 3.** Hazard ratios (HRs) and 95% confidence intervals (CIs) for the association between method of detection and disease-free interval in the 2005 and 2006-2008 cohort^a^

Method of detection	Unadjusted HR (95% CI)^b^	Lead time adjusted HR (95% CI)	Confounding adjusted HR (95% CI)^b^^,^^c^
2005 cohort			
Clinically detected	1.00 (referent)	1.00 (referent)	1.00 (referent)
Screen-detected	0.56 (0.50 to 0.63)	0.77 (0.68 to 0.87)^d^	0.72 (0.64 to 0.81)
2006-2008 cohort			
Nonscreen-related	1.00 (referent)	1.00 (referent)	1.00 (referent)
Screen-detected	0.46 (0.41 to 0.52)	0.76 (0.66 to 0.88)^e^	0.65 (0.57 to 0.73)
Interval	0.88 (0.78 to 0.99)	—^f^	0.83 (0.74 to 0.94)

a All analyses are adjusted for age. Disease-free interval: free of locoregional recurrence, distant metastasis, or contralateral invasive breast cancer.

b Using the uncorrected disease-free interval (ie, time between diagnosis of the primary tumor and diagnosis of the recurrent disease or the end of follow-up).

c Adjusted for age, social economic status, histology, tumor grade, multifocality, tumor stage, and subtype.

d Using the lead time corrected disease-free interval of 6.1 years.

e Using the lead time corrected disease-free interval of 2.0 years.

f Not applicable for this subgroup of patients.

### 2006-2008 cohort

During the 5-year follow-up period, 91.9% of the women with a screen-detected cancer had a 5-year disease-free interval compared with 85.5% of the women with an interval cancer and 83.8% of the women with a nonscreen-related cancer (Figure 2, B). After correcting for lead time, 94.3% of the women with a screen-detected cancer had a 2-year disease-free interval. To compare, 93.6% of the women with an interval cancer had a 2-year disease-free interval and 92.8% of the women with a nonscreen-related cancer. The unadjusted analyses showed that women with a screen-detected or an interval cancer had an improved 5-year disease-free interval compared with women with a nonscreen-related cancer (HR = 0.46, 95% CI = 0.41 to 0.52; HR = 0.88, 95% CI = 0.78 to 0.99, respectively) (Table 3). After correcting for lead time, women with a screen-detected cancer still had an improved 5-year disease-free interval (HR = 0.76, 95% CI = 0.66 to 0.88). After correcting for confounders, and not for lead time, women with a screen-detected or interval cancer had an improved 5-year disease-free interval compared with women with a nonscreen-related cancer (HR = 0.65, 95% CI = 0.57 to 0.73; HR = 0.83, 95% CI = 0.74 to 0.94, respectively) (Table 3;Supplementary Table 2, available online). After adjusting for lead time, women with a screen-detected cancer detected at subsequent screens had an improved disease-free interval compared with women with a nonscreen-related tumor. A similar trend was observed for women with a screen-detected cancer at an initial screen (Supplementary Table 2, available online). After adjusting for confounders, women with a screen-detected cancer detected at initial or subsequent screens had an improved disease-free interval compared with women with a nonscreen-related tumor, as had women with an interval cancer detected after a subsequent screen. A similar trend was observed for women with an interval cancer detected at an initial screen.

## Discussion

This study showed that women with a screen-detected cancer and 10 years of follow-up had an improved disease-free interval compared with women with a clinically detected cancer, taking lead time into account. Moreover, women with a screen-detected cancer or an interval cancer and 5 years of follow-up had an improved disease-free interval compared with women with a nonscreen-related cancer. Our results suggest a positive effect of the screening program.

Tumor characteristics are well known and usually used as confounding factors, as a proxy for correcting for lead time. We showed that after correcting for confounders only, the disease-free interval of the different method of detection groups was of similar order of magnitude as the disease-free interval corrected for lead time in both cohorts. Thereby, our results add more insight to the results of studies that correct for tumor characteristics only. When a reliable estimate of sojourn time is available for a population, we prefer the method correcting for lead time. If this estimate is not available, correcting for confounders (without lead time) can be a good alternative.

In our study, with disease-free interval as outcome, we adjusted for lead time using the Duffy method (8). This has not been described before. Other studies described an advantage in breast cancer–specific survival after lead time correction, which are in accordance with the advantage in disease-free interval described in this study. A British retrospective study, including approximately 27 000 patients, corrected for lead time with a mean sojourn time of 4 years. They found that screen-detected cancer had an increased breast cancer–specific survival (HR = 0.40, 95% CI = 0.37 to 0.44) compared with clinically detected cancer (9). A French study from the region Gironde also showed a statistically increased net survival for screen-detected cancer compared with clinically detected cancer (93.0% vs 83.8%, respectively) after adjustment for lead time (22). Furthermore, we compared our results on disease-free interval correcting for confounders (patient and tumor characteristics) only, with other studies correcting for confounders. Results were similar for the uncorrected and corrected comparison of screen-detected cancers with clinically detected cancers (2,5). Our results support previous studies that showed that method of detection is an independent prognostic factor (23,24). As the results of both methods used in our study are in accordance with previously published studies, this might be a first indication that our lead time–corrected results regarding disease-free interval might be generalizable to other populations. Other studies on method of detection and disease-free interval, correcting for lead time, are needed to support our findings.

Analyzing screen-detected cancers diagnosed at initial or at subsequent screening examinations separately showed that screening seemed to improve the disease-free interval in both situations compared with nonscreen-related cancers. This suggests that the cancers detected at initial screens, which often have worse characteristics compared with cancers detected at subsequent screens (14,25), still have a better prognosis than nonscreen-related cancers. Interval cancers diagnosed after initial or subsequent screening examinations seemed to have an improved disease-free interval compared with nonscreen-related cancers. Even though women are relatively younger at their initial screen compared with at their subsequent screen, the positive effect of the screening program seems to be present in both first and subsequent screens. A possible explanation for the improved disease-free interval of women with an interval cancer compared with those with nonscreen-related cancers is that women who agree to participate in screening might be more conscious about changes in the breast compared with women who do not participate.

In our study, we defined patients with nonscreen-related cancer as a reference group. Women with a screen-detected cancer could also be compared with women with an interval cancer, avoiding self-selection bias regarding attending the screening program. Women with an interval cancer had a statistically worse 5-year disease-free interval compared with screen-detected cancers (results not shown), suggesting a positive effect of the screening program. So far, we found no other studies on the effect of method of detection and disease-free interval. However, the above-mentioned British study (9) also found that screen-detected cancer had an increased breast cancer–specific survival (HR = 0.53, 95% CI = 0.49 to 0.59) compared with interval cancer, which supports our results.

Previous studies showed an improved breast cancer–specific (26) or overall survival (27,28) for patients with a longer time period between breast cancer diagnosis and recurrent disease, or no recurrent disease at all, compared with patients with early recurrence. Although we did not study the association between method of detection and (breast cancer–specific) survival, the improved disease-free interval for screen-detected cancers might suggest improved (breast cancer–specific) survival.

We assumed an average sojourn time of 4.3 years over all breast cancers, which was specific for the population in the region Nijmegen of the Netherlands and a biennial screening program (18). The lead time corrections were based on this average sojourn time. The sojourn time of 4.3 years is the top estimate we found in literature. This relatively high estimate was used, so that any protective effects of screening found would not be due to using a too short sojourn time. When interpreting our results, one should take into account that a screening program with less frequent screening or a different age range included for screening would have a different sojourn time. In addition, the same mean sojourn time was used for every patient, although it is likely that sojourn time differs per patient.

Lead time and length time bias are a concern when comparing survival between different methods of detection groups. Lead time bias has been shown to be decreased by adjusting for tumor size and lymph node involvement (29), while histology and tumor grade have been shown to decrease the effect of length time bias (2,30). Therefore, we performed our analyses correcting for lead time and correcting for confounders separately. Unfortunately, both correction methods used in our study are not the desired gold standard. We realize that our method using lead time correction can be improved when more information becomes available on the length and distribution of sojourn time for individual patients. On the other hand, after correction for confounders, residual confounding might remain.

As treatment options have improved since the introduction of the screening program, the impact of screening on mortality in a recent era remains a point of discussion. A recent meta-analysis showed that most trials and studies found no gain in all-cause mortality due to screening (31). This might suggest that improvements in treatment may have reduced the impact of screening on mortality. Furthermore, all-cause mortality includes a vast number of causes of death on which screening has no effect (32), and others have considered it as a misleading endpoint (33,34). Our study, set in the Dutch screening situation, showed an improved disease-specific interval, corrected for lead time, suggesting that early detection might remain beneficial.

Screening has a strong interaction with early treatment. It should be acknowledged that the benefits of screening described in this study are partly explained by the benefits of early treatment. However, without screening, this early treatment would probably not have been given, and hence there might not have been an improved disease-free interval. A modeling study performed in the United States estimated that, compared with a situation with no screening and no treatment, 37% of the reduction in breast cancer mortality was associated with screening and 63% with treatment in 2012 (35).

Strengths of this study were its nationwide and population-based design, large sample size, and the availability of data on method of detection (because of linkage with the screening program). Also, we were able to study the effect of method of detection on disease-free interval in 2 cohorts (ie, with 5 years or 10 years follow-up available). Furthermore, we were able to correct for lead time, but we could also use well-known tumor characteristics to correct for confounders. Comparing the results of the 2 methods can give additional insight in the size of the risk estimates. Limitations of this study are that the exact sojourn time is not known at the individual level, and the average sojourn time of 4.3 years was thought to be the most suitable time according to a previous Dutch study (18). Finally, length time bias and overdiagnosis might still affect our results.

To conclude, women with a screen-detected cancer had an improved disease-free interval compared with women with a clinically detected cancer, taking lead time into account. More detailed data on method of detection showed that disease-free interval was also improved for screen-detected compared with nonscreen-related cancer. Women with an interval cancer also had an improved disease-free interval compared with nonscreen-related cancer, though this was less pronounced. Correcting for confounders led to results of a similar order of magnitude as correction for lead time. The results of this study suggest that patients with screen-detected breast cancer might have a better prognosis.

## Supplementary Material

djad230_Supplementary_Data

## Data Availability

The data underlying this article cannot be made publicly available because of the privacy of the individuals included in this study. All data collected for the study, including a data dictionary defining each field in the set, will be made available via the NCR (https://iknl.nl/en/ncr/apply-for-data) upon request and after approval of a proposal from the date of publication. The plan for the statistical analysis will be made available by the corresponding author upon request.
