# Supplementary material for: Method of primary breast cancer detection and the disease-free interval, adjusting for lead time
Source: J Natl Cancer Inst. 2023 Nov 3;116(3):370–8. doi: 10.1093/jnci/djad230 (PMC10919328; doi:10.1093/jnci/djad230)
Supplement: djad230_Supplementary_Data [file djad230_supplementary_data.pdf]

Method of primary breast cancer detection and the disease free interval, adjusting for lead time

Linda de Munck, Ph.D.[1]*, Anouk H Eijkelboom, MSc.[1,2]*, Johannes DM Otten, Ph.D.[3], Mireille JM Broeders, Ph.D.[3,4], Sabine Siesling, Ph.D.[1,2]

*These authors have contributed equally to this work and share first authorship

Supplementary Methods

The method of Duffy et al.[8] was used to correct for lead time.
Lead time, i.e. E(s),was calculated according to formula 1 and 2 for each patient with a screen-detected tumor (SDC) using a mean sojourn time of 4.3 years. This mean sojourn time, represents the preclinical screen-detectable period. To correct for lead time, the expected additional follow-up time, i.e. E(s), was subsequently subtracted from the observed disease free interval (DFI) to obtain a corrected DFI for each woman.

According to the method of Duffy et al., women in the 2005 cohort with a SDC, who were event-free after 10-years of follow-up, have a maximum corrected DFI of 6.1 years (10-((1-e-(1/4.3*10))/(1/4.3))). The maximum corrected follow-up time was therefore set at 6.1 years for all women with a SDC. Women with a CDC were censored after 10.0 years. The maximum corrected DFI for women with a SDC who were event-free after 5-years of follow-up was 2.0 years (5-((1-e-(1/4.3*5))/(1/4.3))) (2006-2008 cohort). Hence, the maximum corrected follow-up time of women with a SDC was set at 2.0 years. Women with an IC or NSC were censored after 5 years. The corrected DFI was used in the Cox models to adjusted for lead time.

Formula 1
Calculation of lead time for a patient with a SDC, who has developed a recurrence at time t after diagnosis:

$$E(s) = \frac{\int_0^t xe^{-\lambda x}dx}{1-e^{-\lambda t}} = \frac{1-e^{-\lambda t} - \lambda te^{-\lambda t}}{\lambda(1-e^{-\lambda t})}.$$

1/λ: mean sojourn time (4.3 in the current study)
λ: rate of transition from asymptomatic to symptomatic disease (1/4.3 in the current study)
t: time after diagnosis
E(s): expected additional follow-up time

Formula 2
Calculation of lead time for a patient with a SDC, who has not developed a recurrence at time t after diagnosis:

$$E(s) = \frac{(1-e^{-\lambda t})\int_0^t xe^{-\lambda x}dx}{1-e^{-\lambda t}} + te^{-\lambda t} = \frac{1-e^{-\lambda t}}{\lambda}.$$

1/λ: mean sojourn time (4.3 in the current study)
λ: rate of transition from asymptomatic to symptomatic disease (1/4.3 in the current study)
t: time after diagnosis (maximum follow-up time was 10 years for the 2005 cohort and 5 years for the 2006-2008 cohort)
E(s): expected additional follow-up time

Supplementary Table 1 Multivariable adjusted hazard ratios (HRs) and 95% confidence intervals (CI) for the association between method of detection and risk of developing a recurrent disease (distant metastasis, local regional recurrence or contralateral invasive breast cancer) in the 2005 and 2006-2008 cohort.

	**2005 cohort**	**2006-2008 cohort**
	**HR (95% CI)**	**HR (95% CI)**
**Method of detection**		
Clinically-detected	1.00 (reference)	–[a]
Screen-detected	0.72 (0.64-0.81)	–[a]
Non-screen-related	–[a]	1.00 (reference)
Screen-detected	–[a]	0.65 (0.57-0.73)
Interval	–[a]	0.83 (0.74-0.94)
**Age** (per year)	1.02 (1.01-1.03)	1.02 (1.01-1.02)
**SES**		
High	1.00 (reference)	1.00 (reference)
Medium	1.00 (0.88-1.15)	1.02 (0.91-1.15)
Low	0.99 (0.86-1.15)	1.11 (0.99-1.26)
**Histology**		
Ductal	1.00 (reference)	1.00 (reference)
Lobular	1.05 (0.88-1.25)	1.18 (1.01-1.38)
Mixed	1.05 (0.80-1.38)	1.04 (0.82-1.32)
Other	0.70 (0.51-0.98)	0.78 (0.62-1.00)
**Tumour grade**		
1	1.00 (reference)	1.00 (reference)
2	1.46 (1.22-1.73)	1.39 (1.19-1.62)
3	1.82 (1.49-2.23)	1.99 (1.68-2.36)
**Multifocality**		
No	1.00 (reference)	1.00 (reference)
Yes	1.40 (1.21-1.62)	1.20 (1.06-1.37)
**Tumour stage**		
I	1.00 (reference)	1.00 (reference)
II	1.02 (0.89-1.17)	1.15 (1.02-1.29)
III	2.74 (2.34-3.20)	3.12 (2.74-3.55)
**Tumour subtype**		
ER+ and/or PR+ and HER2-	1.00 (reference)	1.00 (reference)
ER+ and/or PR+ and HER2+	0.88 (0.71-1.10)	1.04 (0.86-1.25)
ER- and PR- and HER2-	1.26 (1.00-1.59)	1.40 (1.17-1.68)
ER- and PR- and HER2+	1.25 (1.02-1.54)	1.86 (1.61-2.16)

ER: estrogen receptor, PR: progesterone receptor, SES: socioeconomic status
[a] Not applicable for this subgroup of patients

Supplementary Table 2 Hazard ratios (HRs) and 95% confidence intervals (CI) for the association between method of detection and disease-free interval in the 2005 and 2006-2008 cohort, stratified by screening round.

	Unadjusted HR (95% CI)[a]	Lead time adjusted HR (95% CI)	Confounding adjusted HR (95% CI)[a,b]
2005-cohort			
Clinically-detected	1.00 (reference)	1.00 (reference)	1.00 (reference)
Screen-detected at initial screening round	0.48 (0.35 to 0.66)	0.65 (0.47 to 0.91)[c]	0.63 (0.46 to 0.87)
Screen-detected at subsequent screening round	0.57 (0.51 to 0.64)	0.78 (0.69 to 0.88)[c]	0.73 (0.64 to 0.82)
2006-2008 cohort			
Non-screen-related	1.00 (reference)	1.00 (reference)	1.00 (reference)
Screen-detected at initial screening round	0.42 (0.32 to 0.56)	0.75 (0.54 to 1.04)[d]	0.61 (0.46 to 0.82)
Screen-detected at subsequent screening round	0.47 (0.41 to 0.52)	0.76 (0.66 to 0.88)[d]	0.65 (0.57 to 0.73)
Interval cancer detected after initial screening round	0.74 (0.56 to 0.99)	–[e]	0.76 (0.57 to 1.01)
Interval cancer detected after subsequent screening round	0.90 (0.79 to 1.02)	–[e]	0.84 (0.74 to 0.96)

All analyses are adjusted for age. Disease-free interval: free of locoregional recurrence, distant metastasis or contralateral invasive breast cancer

[a] Using the uncorrected disease-free interval (i.e. time between diagnosis of the primary tumor and diagnosis of the recurrent disease or the end of follow-up)

[b] Adjusted for age, social economic status, histology, tumour grade, multifocality, tumour stage, and subtype.

[c] Using the lead time corrected disease-free interval of 6.1 years

[d] Using the lead time corrected disease-free interval of 2.0 years

[e] Not applicable for this subgroup of patients
